# Histone Modifications and their Role in Colorectal Cancer (Review)

**DOI:** 10.1007/s12253-019-00663-8

**Published:** 2019-05-04

**Authors:** Jingchun Qin, Bin Wen, Yuqi Liang, Weitao Yu, Huixuan Li

**Affiliations:** 1 Institute of Spleen and Stomach, Guangzhou University of Chinese Medicine, Guangzhou, 510000 China; 2 Lianyungang Affiliated Hospital of Nanjing University of Traditional Chinese Medicine, Nanjing, China

**Keywords:** Histone modification, Colorectal cancer, Histone acetylation, Histone methylation, Histone phosphorylation

## Abstract

The development of colorectal cancer is a complex and multistep process mediated by a variety of factors including the dysregulation of genetic and epigenetic under the influence of microenvironment. It is evident that epigenetics that affects gene activity and expression has been recognized as a critical role in the carcinogenesis. Aside from DNA methylation, miRNA level, and genomic imprinting, histone modification is increasingly recognized as an essential mechanism underlying the occurrence and development of colorectal cancer. Aberrant regulation of histone modification like acetylation, methylation and phosphorylation levels on specific residues is implicated in a wide spectrum of cancers, including colorectal cancer. In addition, as this process is reversible and accompanied by a plethora of deregulated enzymes, inhibiting those histone-modifying enzymes activity and regulating its level has been thought of as a potential path for tumor therapy. This review provides insight into the basic information of histone modification and its application in the colorectal cancer treatment, thereby offering new potential targets for treatment of colorectal cancer.

## Introduction

The accumulation of genetic and epigenetic dysregulation are two kinds of separate mechanisms which resulted in tumorigenesis. Genetic variations have traditionally been regarded as an important player in the occurrence and development of the tumor. Nevertheless, current data have been accumulating concerning the epigenetic change which is responsible for the genesis and progression of cancer [1]. epigenetics is defined as the study of changes in the expression and regulation of genes does not involved in changes in the DNA sequence which can be classified into DNA methylation, histone modification, miRNA, genomic imprinting and chromosome remodeling [2, 3]. Up to now, DNA methylation and histone modification are the most intensively studied epigenetics; Moreover, Histone modification is one of the important regulatory mechanisms of epigenetics, which is detected primarily in the amino- and carboxy-terminal histone tails. It is also an important mechanism in cancer progression [4]. In this setting, a variety of cancer such as gastric cancer, prostate cancer, lung cancer, et al. have been extensively reported to be correlated with this change [5–7]. Colorectal cancer (CRC) is a multi-factorial disease and retains its status as the most common cause of cancer-related death, with more than 6000,000 estimated death across the globe every year [8]. The etiology of colorectal cancer is not completely established, accordingly, it is critical to probe into the molecular bases of colorectal cancer and markers for the early diagnosis and treatment of this cancer. Aberration in histone modification may become an early diagnosis of colorectal cancer. This review focus on understanding the role of histone modifications in the progression of colorectal cancer and their application in colorectal cancer may provide a new direction for diagnosis, treatment,and prognosis of colorectal cancer.

## Histone Modification

**Fig. 1:**
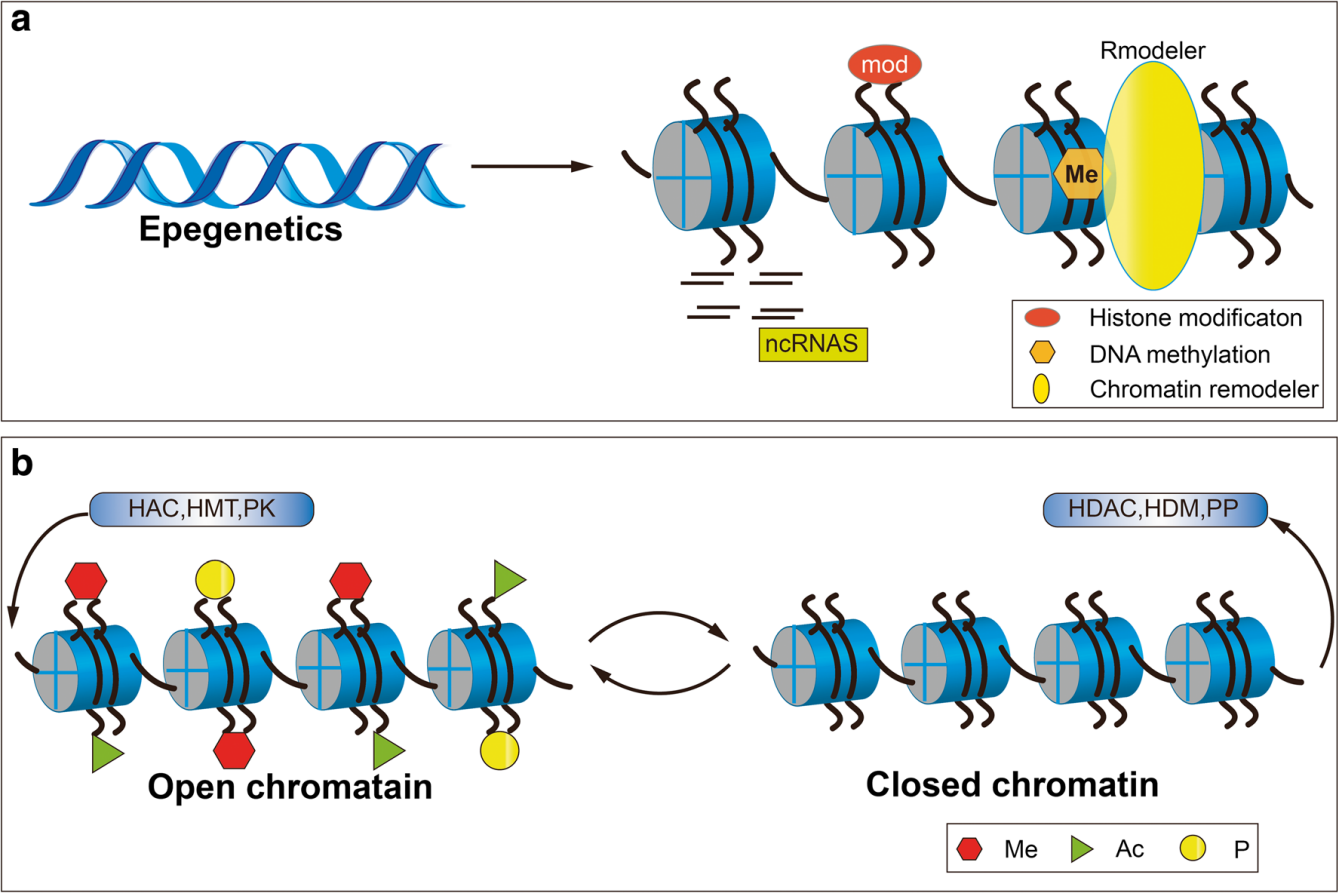
Epigenetics regulate the gene expression without alteration in DNA sequence. It mainly consists of DNA methylation, histone modification, non-coding RNA, chromatin remodeling. **a** The protruding of amino tails of histone modification can undergo several post-translational modifications that affect the expression of genes. Illustrated is the major histone modifications that have been studied in the setting of colorectal cancer such as acetylation, methylation, and phosphorylation. This process is mediated by HATs, HDACs, HMTs, HDMs and protein kinases (PKs). In open chromatin state, histone tail recruits HATs, HMTs, and PKs which can promote gene transcription. In closed chromatin state, histone tail removes these histone-modifying enzymes which can inhibit the gene transcription. (**b**)

Chromatin is a dynamic molecule with multiple structures and there are two basic forms: heterochromatin and euchromatin. Epigenetic events are essential to regulate the condensation state of chromatin and hence to regulate the function of genome by means of histone modifications, chromatin remodeling, DNA methylation and no-coding RNA [9] Fig. 1. the fundamental unit of chromatin is nucleosome, which is composed of two copies of histones H2A, H2B, H3, and H4, wrapping with approximately 147 base pairs of DNA [10]. Histone, one of the major components of chromatin, has long tails protruding away from the nucleosome, which is susceptible to covalently modified at several places [11]. It includes many kinds of covalent modifications, such as acetylation, phosphorylation, methylation, ubiquitylation, sumoylation and so on [12]. At present, more efforts have been put into two aspects of histone modification namely, acetylation, methylation in terms of the orchestration chromatin structure and gene expression [13, 14]. The combination of single or multi-histone modification can interact with each other which forms “histone modification code” [15]. Covalent modification of histones can change the nucleosomal conformation in such a way that modulate the chromatin structure and expression of genes [16]. currently, an emerging role of aberrant histone modifications have been found in a wide spectrum of cancer, giving rise to the role of histone modification is under extensively investigation [13]. The reason why these aberrant modifications of histone lie in inappropriate targeting of histone-modifying enzymes, including HATs, HDACs, HMTs, and HDMTS, locally at gene promoters, resulting in perturbations or mutations in genes [17].

### Histone Acetylation

Reversible histone acetylation is a dynamic process that is achieved by the addition or removal of histone acetyltransferases (HATs) and deacetylases (HDACs) [18]. In recent years, the HATs that has been identified mainly includes P300/CBP, GNAT, MYST, P160, PCAF, TAFII230 families [19]. In the higher eukaryotes, HDACs can be classified into four groups on the basis of their homology with the original yeast enzyme sequence. Class I HDACs, including HDAC1, 2, 3, 8; Class II HDACs composed of HDAC4, 5, 6, 7, 9, 10; Class III HDACs, also known as Sirtuins, comprised of SIRT1–7; Class IV HDACs, including HDAC11 [20]. Among these HDACs Class I, II and IV are zinc-dependent, while Class III are NAD^+^-dependent. HATs transfer the acetyl group of acetyl coenzyme A to the terminal of histone amino acid and relax the structure of chromatin under the action of electric charge, which is helpful to transcription by means of increasing the accessibility of DNA. On the contrary, HDAC removes the terminal acetyl group of histone lysine making the structure of chromatin is compact which result in the inhibition of transcription [21]. In generally, hyperacetylation leads to the increased expression of the gene which is related to the activation of gene transcription, while hypoacetylation means repression of gene expression [22].

### Histone Methylation

Histone methylation refers to the transfer of methyl group to the residues of histone arginine or lysine by taking S-adenosylmethionine as methyl donor under the action of histone methyltransferases (HMTs). Similar to histone acetylation, histone methylation is also a dynamic event which is regulated by different types of HMTs and histone demethytransferases (HDMTs). Histone methylation predominantly occurs in arginine and lysine residues of histone tails and is catalyzed by HMTs. Lysine can be monomethylated, dimethylated or trimethylated, respectively, arginine is either monomethylated or dimethylated. Furthermore, Lysine methylation is a more stable and complex modification of gene expression regulation, which occurs mainly on histone H3 and H4. There are six sites of lysine methylation that have been extensively studied such as H3K9, H3K27, H3K36, H3K79, and H4K20 [23] (Table 1). Protein arginine methylation is chiefly catalyzed by some members of the protein arginine methyltransferase family including PRMT1, PRMT3, PRMT1/HMTA, PRMT4/CARMA, PRMT5 [24]. HMTs comprised of histone arginine methyltransferase (PRMTs) and histone lysine methyltransferase (HKMTs). PRMTs can be classified as two types: Type I catalyzes the formation of mono-methylarginine and asymmetric di-methylarginine; Type II catalyzes the formation of monomethyl arginine and symmetric di-methylarginine. HKMTs mainly include Suv39h1, Suv39h2, G9a, EZH2, SET1, SET2, SET9 which contains the SET domain. DOT1L is the only lysine methyltransferase without the SET domain, which can specifically catalyze the methylation of H3K79 [25]. In addition to the histone methyltransferase, the finding of HDMTs by Shi et al., making the process of histone methylation more dynamic [26]. From then on, a variety of HDMTs has been observed, such as LSD1, UTX, JMJD3, JMJD1A. In contrast to histone acetylation that promotes gene expression, histone lysine methylation has a more complicated effect on gene expression which is depending on the location of modified residues being methylated lead to either transcriptionally-active or -repressive. For instance, methylation at H3K4 and H3K36 sites activate gene transcription. Conversely, methylation at H3K9, H3K27, H3K79, H4K20 sites inhibits gene transcription [27] .

**Table 1:** Classification of histone methyltransferases and demethyltransferases

Class			Members
HMTs	PRMTs	Type I	PRM1, PRM3, PMT1/HMT, PRMT4/CAMR1
		Type II	PRMT5
	HKMTs	Type I	G9a, EZH2, Suv39h1, Suv39h2, SET1, SET2, KMT2A
		Type II	DOTL1
HDMs	LSD1, UTX, JMJD3, JMJD1A		

Adapted from Ref. [23]

PRMTs type I: mono-methylarginine and asymmetric dimethylargine; PRMTs type II: mono-methylarginine and symmeric dimethylarginine; HKMTs type I: lysine-specific SET histone transferases; HMTs type II: without lysine SET histone transferases

### Histone Phosphorylation

Histone phosphorylation, taking place mainly at serines (S), threonines (T) and tyrosine (Y) residues of histone tails, is one of the histone modifications. Phosphorylation disrupts the interaction between histones and DNA attributed to the instability of chromatin structure which is requirement for the structural recombination of chromatin agglutination into homologous chromosomes during mitosis. It matters that different types of the phosphorylated site within histone is intimately linked to different chromatin functions. Moreover, Histone phosphorylation together with other modifications such as histone acetylation is involved in gene transcription, DNA repair, apoptosis and chromosome condensation. For instance, ChIP sequencing data manifested that histone H3 phosphorylated at tyrosine 41 (H3Y41) presents in transcriptional start sites (TSS) function together with H3K4me3 which implicated in transcriptional activation [28]. Furthermore, the combination of H3Y41 and H3K56 function together can significantly increase the accessibility of DNA by more than an order of magnitude [29]. Histone H3 phosphorylated at threonines45 (H3T45) takes part in apoptosis and DNA replication and can promote the acetylation of H3K56 [30, 31]. There is various kinase responsible for phosphorylation such as Aurora kinase(AKs), protein kinase B (PKB/Akt), cyclin-dependent kinases (CDKs), protein kinase C (PKC), casein kinase 2 (Ck2) and Rad3 related kinase ATR [32], and so on. H3Y41 is catalyzed by JAK2 tyrosine kinase (JAK2) [33]. Bub1 is the kinase responsible for phosphorylation of H2AT120 and H3T3 phosphorylated by Haspin kinase [34, 35].

## Histone Modification and Colorectal Cancer

Given that previous research on histone modification in the development of colorectal cancer, the relationship between histone modification and colorectal cancer have been evolving understanding. Dysfunction of histone modification patterns involved in the activation of oncogenes and silence tumor suppressor genes have been verified correlated with the etiology of a variety of human diseases including allergic diseases, multiple sclerosis as well as gastrointestinal cancer [36–38]. Moreover, the alteration of histone modification patterns led to the deregulation of gene expression which plays pivotal roles in the formation of colorectal cancer (Table 2). Therefore, It’s important to investigate the mechanism and biological function of histone modification in CRC, thereby improving the clinical diagnosis and therapy of colorectal cancer.

**Table 2:** Histone acetylation/methylation/phosphory marks in CRC

Modification and sites	Method	Impaired function	References
Histone acetylation marks			
Global H3ac	CHIP, WB	Hyperacetylation (CRC tissues)	[39]
Global H4ac	IHC	Hypoacetylayion (CRC cell lines) induced by CPERT	[40]
H3K9ac	IHC	Hypoacetylation (CRC liver metastasis)	[41]
H3K18ac	IHC	Hypoactylation (CRC cell lines)	[42]
H3K27ac	MS,WB	Hyperacetylation (CRC tissues)	[43]
H3K56ac	WB, CHIP RT-qPCR	Hypoacetylation (CRC cell lines) through RAS-PI3K signal pathway.	[44]
H4K12ac	IHC	Hypoacetylation (CRC cell lines)	[42]
H4K16ac	LC-ES/MS IHC	Hypoacetylation (CRC cell lines, CRC primary tumors)	[45, 46]
Histone methylation marks			
H3K4me2	CHIP, WB	Hypermethylation (CRC tissues)	[39]
H3K4me3	IHC	Hypomethylation (CRC tissues)	[46]
H3K9me2	IHC, WB	Hypermethylation (CRC cells line, CRC liver metastasis)	[41, 47]
H3K27me2	IHC	Hypermethylation (CRC tissues)	[48]
H3K27me3	IHC	Hypermethylation (CRC tissues)	[49]
H3K36me2	IHC	Hypomethylation (CRC liver metastasis)	[48]
H3K79me2	IHC	Hypermethylation (Patient with CRC)	[50]
H4K20me2	LC-ES/MS	Hypomethylation (CRC cell lines)	[45]
H4K20me3	CHIP, PCR	Hypomethylation (CRC patient’s plasma)	[51]
Histone phosphorylation marks			
H3S10ph	IHC	Hypophosphorylation (CRC cell lines)	[52]
H2AX	IHC	Hyperphosphorylation (CRC patients)	[53]

### Histone Acetylation and Colorectal Cancer

Up to now, it has been the overwhelming accumulation of evidence indicated that histone acetylation finely regulates a wide range of cell functions, such as cell differentiation, assembly of the nucleosome, the change of chromatin structure and stability of gene expression [43]. it’s not surprising that abnormal regulation of histone acetylation is relevance to the predisposition to developing colorectal cancer and this process is modulated by a plethora of deregulated enzymes including HATs, HDATs. Karczmarski et al. used both mass spectrometry (MS) and western blot showed that acetylation of H3K27 was significantly increased in CRC samples compared with normal tissue [42]. Ashktorab et al. reported that acetylation of H3K12ac and H3K18ac was significantly increased in moderate to well differentiated colonic cancer, whereas decreased in poorly differentiated colonic cancer. They also observed that high level of HDAC2 in adenocarcinoma compared with those in adenoma, suggesting that the expression of HDAC2 is closely related to the progression from adenoma to adenocarcinoma [45]. Fraga et al. demonstrated that colorectal cancer is accompanied by reduced histone acetylation on H4K16 in CRC cell lines used both LC-ES-MS and western blot [54]. furthermore, several reports has indicated that global histone acetylation was positively correlated with tumor stage, lymph metastasis, poor survival, poor prognosis, histological subtype and cancer recurrence [17, 39]. Hashimoto et al. uesd multivariate analysis found that up-regulated global expression level of acetylated histone H3 (H3Ac) in colorectal cancer tissues was linked to poor overall survival. in addition, high AS (Allred scoring system) score of H3Ac predicted poor prognosis [41]. Tanagawa et al. demonstrated that global hypoacetylation of H3K9 was significantly associated with the histological type of colorectal cancer using immunohistochemistry [46]. Benard et al. found that increased nuclear expression of H3K56ac and H4K16ac was highly correlations with better survival of CRC patients and a lower chance of tumor recurrence [55].

Recent intensively investigations in several of cancers focus on altering expression of HATs or HDACs uncovered that they contribute to tumorigenesis. Research data have indicated that low expression of males absent on the first (MOF) appeared in colorectal cancer and it mainly correlated with lymph node metastasis and tumor stage in patients with CRC [56, 57]. Previous studies indicated that CLASS I HDACs are generally up-regulated in normal colon tissues and colon cancer cell lines [58, 59]. HDAC1 was shown to higher in CRC tissues than in normal tissues and low expression of it indicated better overall survival (OS) [60]. HDAC2 has been found up-regulated in CRC cell lines as compared with their corresponding normal colonic epithelial cells [61]. Nemati et al. used RT-PCR observed that an increased level of HDAC3 in CRC specimens in relation to poor tumor differentiation [62]. In addition, there is also some study indicated that certain histone acetylation can be targeted via specific signaling pathway [44]. For example, Liu et al. discovered that RAS-PI3K signaling down-regulates the level of H3K56ac which is related to transcription, proliferation, and migration of cancer cells [40]. The study by Zhang et al. uncovered that cell-cycle related and expression-elevated protein (CREPT) cooperated with acetyltransferase P300 stimulates the Wnt/−catenin signaling to promote the expression of H4Ac and H3K27ac [47].

### Histone Methylation and Colorectal Cancer

Histone methylation is involved in diverse biological functions including the formation of heterochromatin, inactivation of the X chromosome, DNA damage response and transcriptional regulation. The abnormal biological function of histone methylation regulates pathogenesis of various diseases including tumors. To date, ectopic expression of histone methylation and demethylases have been widely described in several cancers including colorectal cancer. For instance, Nakazawa et al. Used both immunohistochemistry and western blot analysis have revealed that increased expression of global level of dimethylated lysine 9 on histone H3 (H3K9me2) in the nuclei of adenocarcinoma more than that in adenoma, suggesting that hyperacetylation of H3K9m2 might be relevant to the adenoma transition to adenocarcinoma [63]. Yokoyama et al. reported that the methylation level of trimethylated lysine 9 on histone 3 (H3K9me3) was especially up-regulated in invasive regions of colorectal cancer tissues and H3K9 trimethylation was positively related to lymph node metastasis. in addition, elevated expression of H3K9 methyltransferase SUV39H1 was facilitated the development of CRC which resulted in a poor survival rate in mouse [51]. while Gezer.et al. uncovered that histone methylation marks H3K9me3 and H4K20me3 was significantly decreased in plasma of the patient with CRC [48]. High AS score of H3K4me2 is dramatically associated with colorectal cancer clinicopathological factors including deeper tumor invasion and advanced pathological stage [41]. What is more, Multivariate survival analysis revealed that the low expression of H3K4me2 could be served as an independent prognostic factor in CRC patients with metachronous liver metastasis [46]. The low expression level of H4K20me2 was a common hallmark in CRC cell lines [54]. Tamagawa et al. showed that decrease methylation of H3K27me2 in liver metastasis in comparison with primary tumors, whereas the expression of H3K36me2 was reversed. They also demonstrated that the expression level of H3K37me2 is positively associated with tumor size and poorer survival rates and it could be served as an independent prognostic factor for CRC patients with metachronous liver metastasis [49]. Benard et al. found that an up-regulated level of H3K27me3 compared to normal counterparts that were detected by immunohistochemically stained (IHC). it closely linked to better patient survival and longer recurrence-free periods [64]. They also observed that increase expression of H3K4me3 and decrease expression of H3K9me3 and H4K20me3 were associated with shorter survival and higher chances of tumor recurrence in the early stage of colon cancer [50]. Additionally, high levels expression of H3K79me2 was suggested to be a predictor of poor CRC patient survival [65].

Global histone methylation is controlled by histone methyltransferase (HMTS) and demethyltransferase (HDMTS) plays an essential role in the regulation of chromatin structure and function. Many recent studies have discovered that the alteration of HATs and HDMTs was documented in different types of cancers [66]. Kornbluht described that reduction of histone methyltransferase SEDT2 facilitated the CRC development by affecting alternative splicing [67]. The study by Qin et al. showed that the expression of G9A was dramatically increased in CRC tumor tissues and overexpression of G9A was mainly correlated with American Joint Committee on Cancer staging (AJCC), tumor differentiation and tumor relapse of CRC [68]. The histone H3K27 methyltransferase EZH2 expression was up-regulated in CRC, which was predicted shorter survival and advanced stage implying that it could use to an indicator of clinical outcome in CRC patients [69, 70]. Low nuclear expression of demethylase JMJD3 was shown in normal colorectal tissues as compared with CRC tissues and low expression of JMJD3 could serve as an independent predictor of poor prognosis in patients with CRC [71]. Elevate expression of Lysine-specific demethylase (LSD1) observed in colon cancer tissues, and high expression level of LSD1 was strongly correlated with advanced TNM stages and distant metastasis [72].

### Histone Phosphorylation and Colorectal Cancer

Histone phosphorylation is essential for maintaining the equilibrium of kinase-phosphatase at kinetochore to refrain from chromosomal instability and cancer. Accordingly, there is an increasing body of investigation evaluating the impact of dysregulated phosphorylation on the development of many human diseases, including colon cancer [73]. As yet, there are few researches address the relationship between histone phosphorylation and colorectal cancer. Several studies have shown that aberrant of phosphorylation histone has been verified to correlated with the pathogenesis of colorectal cancer. For example, downregulation of dual specificity phosphatase 22 (DUSP22) expression was observed in colorectal cancer specimens and reduced DUSP22 expression in stage IV patients was mainly exhibited poor survival outcome [53]. Lee et al. revealed that phosphorylation of the H2AX histone (p-H2AX) have been found elevated in CRC tissues and have been corrected with a more aggressive type of tumor behavior, as well as poor CRC patient survival [74]. Chen et al. found that PKCƐ modulated MllP-S303 phosphorylation and its expression level was associated with metastasis and prognosis of colorectal cancer [52]. Xiao et al. identified that a reduced level of Histone H3 at Ser10 (H3S10) was observed in colon cancer. Meanwhile, the phosphorylation of T-LAK cell-originated protein kinase (TOPK) at Y74 and Y272 facilitated the carcinogenesis of colon cancer [75].

## Application of Histone Modification in Colorectal Cancer

Conventional CRC therapies generally including chemotherapy, surgery and radiation therapy. However, clinic efficacy of those treatments is limited. Recent investigation has revealed that the process of histone modification is reversible and their aberrations can be restored to nearly normal status through epigenetic therapy. Thus, histone modification serves as a promising therapeutic target in treating various cancers in combination with conventional treatment. Histone deacetylation and methylation inhibitors are the most widely applied to colorectal cancer.

### Histone Methyltransferase Inhibitors and Treatment of Colorectal Cancer

Previous studies have emphasized the significance of histone methylation in the regulation of gene and other physiological processes. Furthermore, aberrant histone methylation as a result of gene mutation is frequently associated with the occurrence and development of cancer. In order to provide a broader platform for cancer treatment, the study communities further identify small molecule inhibitors targeting either histone methyltransferases or demethylases for the therapy of CRC, therefore most of the histone modifying enzymes serve as a drug target has been widely reported [76]. The study by Hsu et al. was reported that LSD1 inhibitors CBB1003 suppress CRC cell growth through down-regulating LGR5 levels and inactivates the Wnt/β-catenin pathway [77]. Enhancer of zeste homolog 2 (EZH2) is a subunit of the polycomb repressive complex 2 (PCR2) and high level of EZH2 has been observed in different cancers including bladder cancer, non-small-cell lung cancer as well as colorectal cancer [78]. Therefore, EZH2-specific inhibitors have been regarded as an appealing target as a result of its oncogenic activities. EZH2 inhibitor GSK346 enjoys good anti-tumor efficacy for it can suppress migration, invasion, and proliferation of CRC cells [79]. Likewise, in vitro investigations have shown that UCN1999 and GSK 343 are two S-adenosyl-L-methionine (SAM) -competitive inhibitors which promoted autophagy through upregulated the expression of LC3 gene resulted in colorectal cancer cell death [80]. Verticillin A, a selective histone methyltransferase inhibitor, not only effectively inhibited the metastatic CRC cell growth but also enhanced the efficacy of CTL immunotherapy to block the progression and metastasis of CRC [81]. Nowadays, in vitro study shows that JIB-04, a novel histone demethylase inhibitors targets colorectal cancer stem cells (CSC), was able to repress CSC growth, invasion, and migration to fight against colorectal cancer [82].

### Histone Deacetylase Inhibitors and the Treatment of Colorectal Cancer

According to HDACis’s molecular function, they can be divided into four major groups: the first group is short-chain fatty acids mainly including phenylbutyrate (PB), valproic acid (VPA) and carboxylic acids NaB. The second group is hydroxamates consisting of TSA and SAHA. The third group is benzamides containing MS-275 and MGCD-0103. the fourth group is cyclic peptides. As an emerging sort of anti-cancer drug, increasing evidence has demonstrated that HDACis exert their anti-tumor effects is conveyed by regulating multiple approaches including induce tumor cells cycle arrest, inhibit tumor cell growth, differentiation apoptosis. Furthermore, they facilitate the acetylation of histone and nonhistone protein resulted in the alteration of their transcriptional activity [83]. they inhibit angiogenesis and modulate the miRNA expression in tumor progression [84]. Major clinic HDACis for the treatment of CRC is summarized in Table 2.

The current investigation indicated that deregulation of HATs and histone HDACs is engaged in the progression of a range of cancers, making them spur the considerable interest of the research community [22, 85]. Thus, HDACis become appealed target in attempts to attenuate many human cancers including colorectal cancer. Therefore, various histone deacetylase inhibitors (HDIs) become the favored target in attempts to attenuate much human cancer including colorectal cancer. So far, there are four HDACis have been approved by Food and Drug Administration (FAD) for the treatment of patients with cutaneous T cell lymphoma and peripheral T cell lymphoma [86, 87]. Even if there is a growing list of HDACis applied to colorectal cancer, a wealth of candidates are ongoing intensively study and clinical trials. For example, Trichostatin A (TSA) suppress the growth of CRC cells in vivo by inducing cell cycle arrest and apoptosis through the modulation of JAK2/STAT3 signaling [88]. SAHA known as suberoylanilide hydroxamic acid inhibits colon tumor growth via decreasing the expression of histone deacetylases, cyclin D1 and survivin [89]. in addition, SAHA exerts their anti-proliferative effects in CRC cells through reducing expression of oncogenic miR17–92 cluster miRNAs [90]. Treating colorectal cancer with Valproate (VPA) could depress tumor growth with cell cycle through alteration of H3 and H4 acetylation [91]. Romidepsin, one of the new class of histone deacetylase inhibitors, exhibits its anti-neoplastic effect in colorectal cancer cell lines via induced alteration in protein modification including acetylation and phosphorylation [92]. In vitro data indicated that it was able to induce apoptosis by the generation of reactive oxygen species (ROS) [93]. Butyrate is a kind of short fatty acid which has been shown to significantly effective against the migration and invasion of CRC cell lines via activating the A kt1 and ERK1/2 signaling [94]. Entinostat (MS-275) is a member of benzamides, in various CRC lines and xenograft models it has been shown potent anti-proliferative effects and reduce tumor volume [95] and it facilitates Natural killer (NK) cell killing of tumor cells by regulation both the NKG2D receptor and its ligand, implying that augment NK cell immunotherapy may be a potential approach for solid tumors [96]. Furthermore, It is recently shown that Dihydroxybenzoic acid (DHBA), a kind of benzoic acid derivatives, can suppress HDAC activity resulted in cancer cell growth inhibition via the induction of ROS and cellular apoptosis regulated by Caspase-3 [97]. In CRC cell lines, Belinostat induces apoptosis and inhibit colon cancer cell proliferation by the regulation of proteins including p53, AP1 [98]. In HCC and CRC cell lines treated with panobinostat (LBH589) was able to reduce proliferation and vascularization lead to a suppressed tumor growth [99], this observation is in accordance with previously published results [100] (Table 3).

**Table 3:** The implication of histone acetylase inhibitors(HDACis) in CRC treatment

Class	Specificity	HDACis	Experiment design	Effect	Reference
Hydroxamic acids	Class I, II	Trichostatin A (TSA)	CRC cell lines	Inhibit CRC cells growth	[87]
		Vorinostat (SAHA)	Mouse model	Inhibit the growth of colon tumors in nude mice	[88]
			CRC cell lines	Anti-proliferative in CRC cell lines	[89]
		Panobistat	CRC cell lines HCC cell lines	Anti-angiogenic Reduce proliferation	[98]
		Belinostat	CRC cell lines	Induce CRC cell apoptosis	[97]
Short-chain fatty acids	Class I	Butyrate	CRC cell lines	Inhibit CRC cell migration and invasion	[93]
		Valproic acid (VPA)	CRC cell lines	Inhibit the growth of CRC cell lines with cell cycle arrest	[90]
Benzamides	Class I	Entinostat (MS-275)	CRC cell lines mouse model	Anti-proliferative effects	[94]
Benzoic acid		Dihydroxy benzoic acid	Colon cell lines	Induce cancer cell death	[96]
Cyclic peptides	Class I, II	Romidepsin (FK228)	CRC cell lines	Anti-tumor activity	[91]

It has been noted HDAC inhibitors as monotherapy were initially incorporated into the clinical exhibits limited effectiveness, acquisition of drug resistance as well as adverse effect in the treatment of cancer [101, 102]. Hence, the tendency to apply them in corporation with different types of anti-cancer drugs is increasing. Several studies show the combined therapies may amplify the anti-tumor effect to suppress refractory tumors [103]. The plethora of investigation involved in anti-tumor agents and HDAC inhibitors synergistically used. For example, A452, an HDAC6-selective inhibitor, in combination with SAHA enhanced anti-proliferation effects on CRC cell lines compared with single-agent therapy [104]. EZH2 inhibitors and EGFR inhibitors synergisticll induced autophagy and apoptosis, resulting in the inhibition of colon cancer cells [105]. Combination treatment with vorinostat and bortezomib has better suppress proliferation and induce CRC cell cycle arrest than treatment with single-agent therapy [106]. The combined use of Butyrate and irinotecan exhibit potentiate the antineoplastic effects in CRC cell lines, resulting in tumor cell death [107]. while combining entinostat with demethylating agent 5-azacitidine applied with metastatic CRC patients have no activity in colorectal cancer [108]. Moreover, a variety of HDAC inhibitors are lack of specificity, thus, it is more critical to devote to developing effective and specific epigenetic targets against colorectal cancer and provide an optimized therapeutic regimen for CRC patients. Accordingly, in future, the current study is primarily concentrates on synergistic effects achieved by the combination use these agents which will represent great promise for further development in human cancer therapy.

## Conclusion and Future Perspectives

In summary, there are plenty of publications with respect to histone modification in colorectal cancer, however, its complete picture remains unclear. This article attempts to comprehensively elucidate the essential role of histone modification in CRC. we are now clear that histone modification is involved in the pathogenesis of CRC. Therefore, it is important for us to apply this new understanding to develop novel therapeutic approaches for cancer. However, there are still some problems. First, the contribution of histone modification dysregulation in colorectal cancer isn’t completely known, hindering the discovery of emerging target for cancer therapy. Second, the different subtypes of CRC caused by emerging abnormal expression pattern of histone modification on specific residues in the progression of CRC that requires specificity of epigenetic drugs to provide individual treatment for CRC patients. Third, considering monotherapy have limited anti-tumor efficacy and lead to adverse effects, combining with other anticancer drugs for the treatment of this malignant neoplasm is absolutely necessary. In the future, with great advances made in this evolving area, the future of epigenetic drug is bright, despite the fact that we have faced these problems. Furthermore, as researcher communities delicate to exploring more advances effective combination inhibitors of epigenetic drugs to alter the course of colorecter cancer. It’s firmly believed that these will bring the benefit to colorectal cancer patients.
